# A case–control genome-wide association study of ADHD discovers a novel association with the tenascin R (*TNR*) gene

**DOI:** 10.1038/s41398-018-0329-x

**Published:** 2018-12-18

**Authors:** Ziarih Hawi, Hannah Yates, Ari Pinar, Aurina Arnatkeviciute, Beth Johnson, Janette Tong, Kealan Pugsley, Callum Dark, Marc Pauper, Marieke Klein, Helen S. Heussler, Harriet Hiscock, Alex Fornito, Jeggan Tiego, Amy Finlay, Alasdair Vance, Michael Gill, Lindsey Kent, Mark A. Bellgrove

**Affiliations:** 1 School of Psychological Sciences and Monash Institute for Cognitive and Clinical Neurosciences (MICCN), Monash University, Melbourne, Australia; 2 Departments of Human Genetics, and Psychiatry, Radboud University Medical Center, Nijmegen, The Netherlands; 3 Mater Research Institute, University of Queensland and Children’s Health Queensland, South Brisbane, Australia; 4 Pediatrics Royal Children’s Hospital, Murdoch Children’s Institute, Melbourne, Australia; 5 The Royal Children’s Hospital, University of Melbourne, Victoria, Australia; 6 Department of Psychiatry, Trinity College, Dublin, Ireland; 7 School of Medicine, University of St Andrews, St. Andrews, Scotland UK

## Abstract

It is well-established that there is a strong genetic contribution to the aetiology of attention deficit hyperactivity disorder (ADHD). Here, we employed a hypothesis-free genome-wide association study (GWAS) design in a sample of 480 clinical childhood ADHD cases and 1208 controls to search for novel genetic risk loci for ADHD. DNA was genotyped using Illumina’s Human Infinium PsychArray-24v1.2., and the data were subsequently imputed to the 1000 Genomes reference panel. Rigorous quality control and pruning of genotypes at both individual subject and single nucleotide polymorphism (SNP) levels was performed. Polygenic risk score (PGRS) analysis revealed that ADHD case–control status was explained by genetic risk for ADHD, but no other major psychiatric disorders. Logistic regression analysis was performed genome-wide to test the association between SNPs and ADHD case–control status. We observed a genome-wide significant association (*p* = 3.15E−08) between ADHD and rs6686722, mapped to the Tenascin R (*TNR*) gene. Members of this gene family are extracellular matrix glycoproteins that play a role in neural cell adhesion and neurite outgrowth. Suggestive evidence of associations with ADHD was observed for an additional 111 SNPs (⩽9.91E−05). Although intriguing, the association between DNA variation in the *TNR* gene and ADHD should be viewed as preliminary given the small sample size of this discovery dataset.

## Introduction

Attention deficit hyperactivity disorder (ADHD) is the most commonly diagnosed neurodevelopmental disorder of childhood. It is typically characterised by a persistent pattern of inattention, impulsivity and/or hyperactivity. Longitudinal studies indicate that approximately 66–77% of individuals who experience childhood ADHD continue to experience at least subthreshold symptoms of ADHD that significantly impact adulthood functioning^1,2^. The global prevalence of ADHD has been estimated at 5.2–7.2%^3,4^. Although the aetiology of ADHD is not well defined, genetic and environmental factors have been implicated in the disorder. Despite minor disparities across individual samples and study designs, the overall heritability of ADHD is estimated at 70–90%^5,6^.

The efficiency of indirect dopamine agonists in reducing the symptoms of ADHD led to the development of the ‘dopamine hypothesis of ADHD’, which postulated that dysregulated dopamine signalling is central to the pathophysiology of ADHD^7^. Putative disruption of other monoamines such as noradrenaline and serotonin in ADHD has also been proposed. Accordingly, genetic markers mapped to these monoamine pathways have historically been pursued as candidate genes for ADHD. Although a number of replicated findings exist within this candidate gene literature^8,9^, a key limitation remains the a priori selection of genes based upon incomplete knowledge of the biology of ADHD, which may ultimately impede the identification of novel risk markers.

Genome-wide association studies (GWAS) allow the identification of novel risk variants without prior knowledge of the biology of a trait or disorder. Further, this approach is aligned with the polygenic nature of complex disorders whereby the small role of individual single nucleotide polymorphisms (SNPs) can be considered in aggregate to better understand how genetic susceptibility may arise. To date, 13 ADHD–GWAS have been published. Of these, seven were case–control studies^10–16^, two were family-based analyses^17,18^, and three examined the association between quantitative ADHD symptom measures and genetic markers^19–21^. A GWAS meta-analysis was also performed in 2010 ^22^. Overall, these previous ADHD–GWAS had limited success in identifying associations. However, preliminary evidence of associations (albeit below GWAS significance) were identified for genes that function in biological processes relevant to ADHD aetiology. For example, pathway analysis highlights a potential role for potassium channel genes and activation Ras Homologue Family Member A (RhoA) signalling genes, lending further support to hypothesised dysregulation of neurotransmitter release in ADHD^23^. Additional pathway analysis utilising data arising from five ADHD–GWAS using the Ingenuity and BiNGO tools, showed significant enrichment of genes mapped to a network involved in neurite outgrowth whose targets are modulated by drugs used to treat ADHD^24^. Most recently, a large meta-analysis of GWAS data arising from 20,183 ADHD cases and 35,191 controls yielded the first 12 independently significant ADHD–GWAS loci^25^.

Here, we conducted a GWAS on a rigorously diagnosed clinical ADHD cohort collected across Australia, England and Ireland in an attempt to clarify further the genetic architecture of ADHD and to potentially identify novel genetic risk factor(s). An additional important purpose of the current study was to contribute to the expansion of the international GWAS community. In this context, GWAS data derived from this study can be combined with those of the international ADHD–GWAS community (e.g., ADHD–PGC) for subsequent GWAS analyses.

## Materials and methods

### Participants

Five hundred and sixty seven (*N* = 567) children with ADHD of European ancestry were recruited from Australia, the United Kingdom and Ireland. None of the ADHD probands were included in previously published GWAS. All cases met the DSM-IV diagnostic criteria for ADHD at the time of sample collection. ADHD status was determined through parental semi-structured interview and completion of the Conners’ Parent Rating Scale for ADHD, which has demonstrated internal reliability and criterion validity for use in assessing ADHD^26^. Children with an IQ less than 70 as determined using the Wechsler Intelligence Scale for Children version 4 (WISC-IV Standardisation Sample, 2003)^27^ were excluded. One thousand two hundred and ninety-six control (*N* = 1296) participants were also recruited. All patients and controls were European by descent based on self-report ethnicity of all four grandparents^28^. All control participants were recruited in Australia and had no self-reported personal history of psychiatric or neurological disorders including ADHD. Written informed consent was provided by the individual in the case of adults, or the primary caregiver or guardian in the case of children/adolescents.

### DNA genotyping and quality control

DNA samples were genotyped using the Illumina Infinium PsychArray-24v1.2 BeadChip at Path West’s Diagnostic Genomics Laboratory in Western Australia. The Illumina Psych-Chip has a backbone of 510,000 markers comprising 265,000 tagging SNPs found on the Infinium Core-24 BeadChip and 245,000 markers from the Infinium Exome-24 BeadChip. It was developed in collaboration with the Psychiatric Genomics Consortium (PGC) and supplemented with an additional ~50,000 SNPs implicated in psychiatric and neurodevelopmental disorders. To avoid spurious GWAS findings, we adopted a stringent quality control (QC) protocol using PLINK 1.9 software at both the individual subject and SNP level^29,30^.

#### Subject-level QC

The following subject-level QC was employed: (1) We initially removed individuals with low-genotyping score by excluding participants with ⩾0.03 of missing data; (2) we performed identity by descent analysis to detect and remove possible sample contaminations, duplications as well as unknown familial relationships (such as relatedness); (3) we applied principal components analysis to identify any potential sources of population stratification and removed outlier subjects; (4) we tested for unusual heterozygosity, which refers to the presence of more or less heterozygous SNPs across the genome than would be expected by the population mean, and removed individuals displaying outlying mean heterozygosity (greater than ±3 SDs from the sample mean); and (5) disparities between recorded and observed sex status were determined through X-chromosome homozygosity were removed.

Following the above rigorous QC, 480 ADHD probands (Australians = 365, English = 62 and Irish = 53) remained in the final set for analysis. All children were aged between 5 and 18 years (age mean; Age_M_ = 10.27 years, Age_SD_ = 3.03). Of the ADHD cases 87% were male and 13% were female. The high frequency of male participants in the ADHD cohort is reflective of the sex ratio in clinical populations^31^. For controls, 1208 participants aged between 7 and 60 years (Age_M_ = 20.61 years, Age_SD_ = 6.76) were carried forward to the final statistical association analysis. Of these participants, 49% were male and 51% were female.

#### SNP-level QC

Prior to imputation, additional filtering steps were conducted by removing 3516 SNPs with genotyping call rate <95. In addition, genotyped SNPs departing from Hardy–Weinberg (H–W) equilibrium were also excluded. This resulted in the removal of 76 SNPs. A further 1855 SNPs with significantly different (*p* ≤ 1.0E−5) missing genotype rates between cases and controls were also removed. Finally, SNPs with a minor allele frequency (MAF) < 0.01 were removed leaving 290,265 in the final set taken forward to imputation.

### DNA imputation

The freely available software packages MaCH and Minimac2 were used for phasing and genotype imputation employing the 1000 Genomes reference panel (hg 19 build 37)^32,33^. Finally, a MAF ⩾ 0.05 was implemented for our final association analysis. These constraints yielded a final set of 5,407,269 SNPs which were subjected to statistical association analysis.

### PGRS calculation

Polygenic risk scores (PGRS) for the five major psychiatric disorders including ADHD, autism spectrum disorder (ASD), schizophrenia (SCZ), bipolar disorder (BD) and major depressive disorder (MDD) were calculated using PRSice software package^34^. PGRS for each subject and disorder were estimated as a sum of risk alleles weighted by their effect size as defined by data arising from the latest publically available GWAS^25,35–38^. For each disorder PGRS were calculated at a 1000 *p* thresholds (*p*_T_) ranging from 0.0005 to 0.5. To find the most predictive *p*_T_ logistic regression was applied at each threshold using ADHD status as a regressor and Nagelkerke’s *R*^2^ and the corresponding *p* values were estimated. Analysis of PGRS here served to demonstrate that the current ADHD cohort replicated the published and publically available genetic risk profile for ADHD.

### ADHD case–control genome-wide association analysis

Association analysis was performed in 480 ADHD cases and 1208 controls using logistic regression analysis implemented in PLINK 1.9. The following covariates were included: age, age^2^, age × gender, and the top five eigenvectors accounting for population stratification.

## Results

### PGRS analysis

**Fig. 1:**
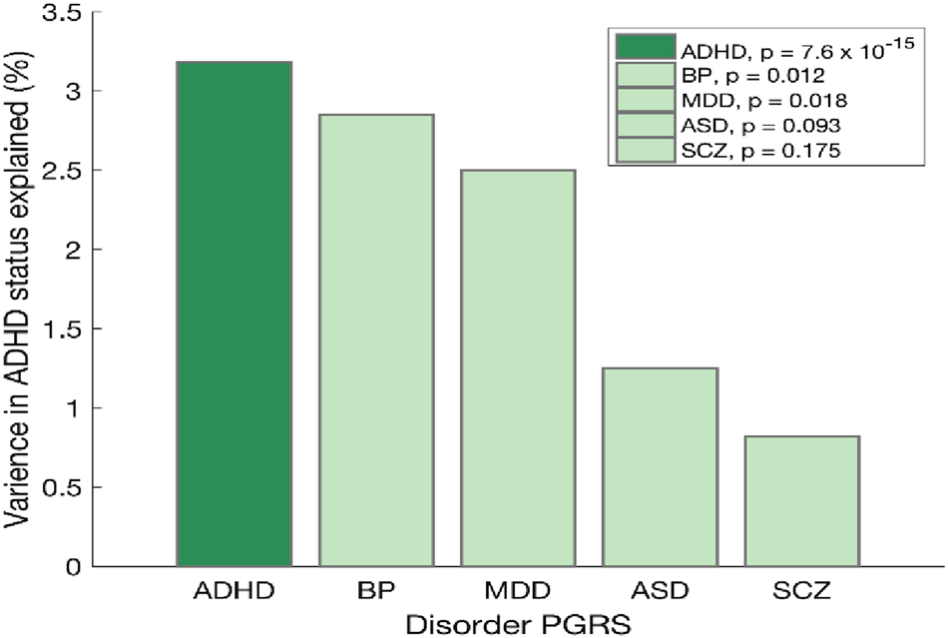
Diagrammatic representation showing the polygenic risk scores (PGRS) for ADHD, BP, MDD, ASD and SCZ against the ADHD case–control statues for the current cohort. Only ADHD–PGRS were significantly predictive of ADHD status (*p* = 7.6 × 10^−15^) explaining 3.25% of variance in the ADHD case–control status

Here, we utilised PGRS analysis to determine whether our ADHD cohort replicated the published ADHD genetic risk profile. Logistic regression was used to examine the relationship between PGRS for each of the 5 major psychiatric disorders and ADHD case–control status, for 1000 *p*_T_ values ranging from 0.0005 to 0.5. Age, gender, age^2^, age × gender, along with the top five eigenvectors accounting for population stratification were used as covariates. As expected, only ADHD PGRS was significantly predictive of ADHD case–control status (Nagelkerke’s *R*^2^ = 0.03, *p* = 7.6E−15 at *p*_T_ = 0.0785). PGRS for the other four psychiatric disorders did not reach the recommended statistical significance threshold of *p* = 0.001at any *p*-threshold (Figure 1)^34^. The best model fit values for the remaining four psychiatric disorders were as follows: BP (Nagelkerke’s *R*^2^ = 0.00285, *p* = 0.012, *p*_T_ = 0.011), MDD (Nagelkerke’s *R*^2^ = 0.00250, *p* = 0.018, *p*_T_ = 0.103), ASD (Nagelkerke’s *R*^2^ = 0.00125, *p* = 0.093, *p*_T_ = 0.1), SCZ (Nagelkerke’s *R*^2^ = 0.00082, *p* = 0.175, *p*_T_ = 0.0335).

### ADHD case–control genome-wide association analysis

**Fig. 2:**
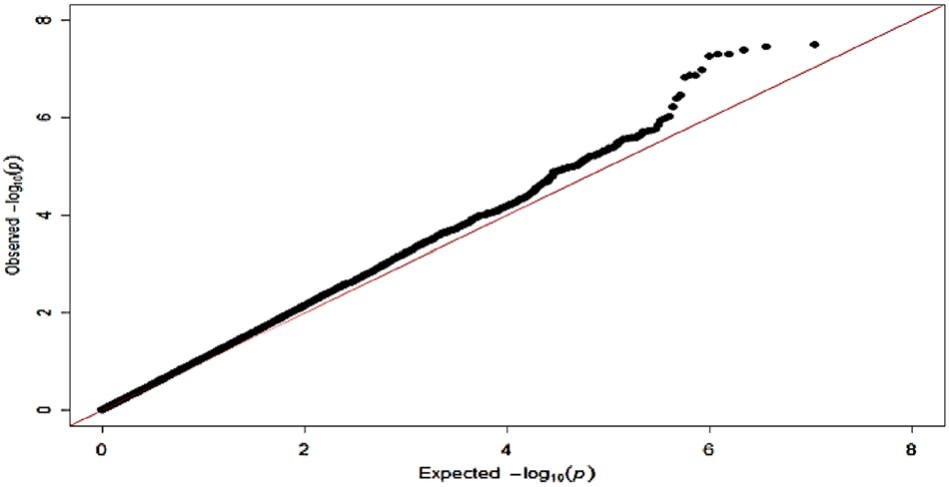
Q–Q plot of ADHD–GWAS using 5,407,269 imputed SNPs A deviation in the observed p-value at the top end consistent with genetic influence

**Fig. 3:**
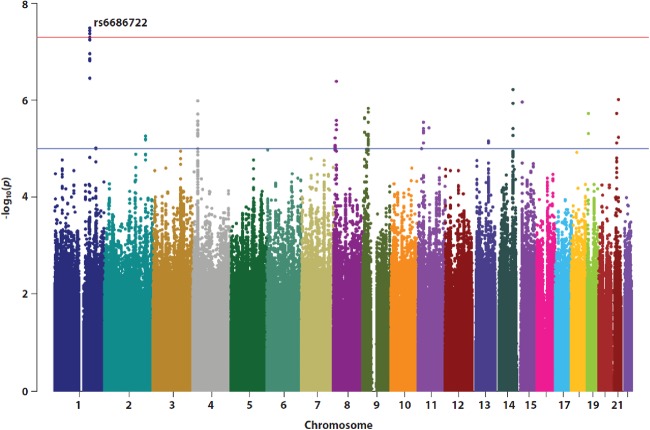
Manhattan plot of the ADHD–GWAS showing −10 log (*p* value) versus genomic location for autosomal chromosomes (1–22). Results show a significant association of rs6686722 on chromosome 1 (*p* = 3.1E−08). The horizontal blue and orange lines represent *p* values at 1.0 × 1E−05 and 5.0E−08, respectively

**Fig. 4:**
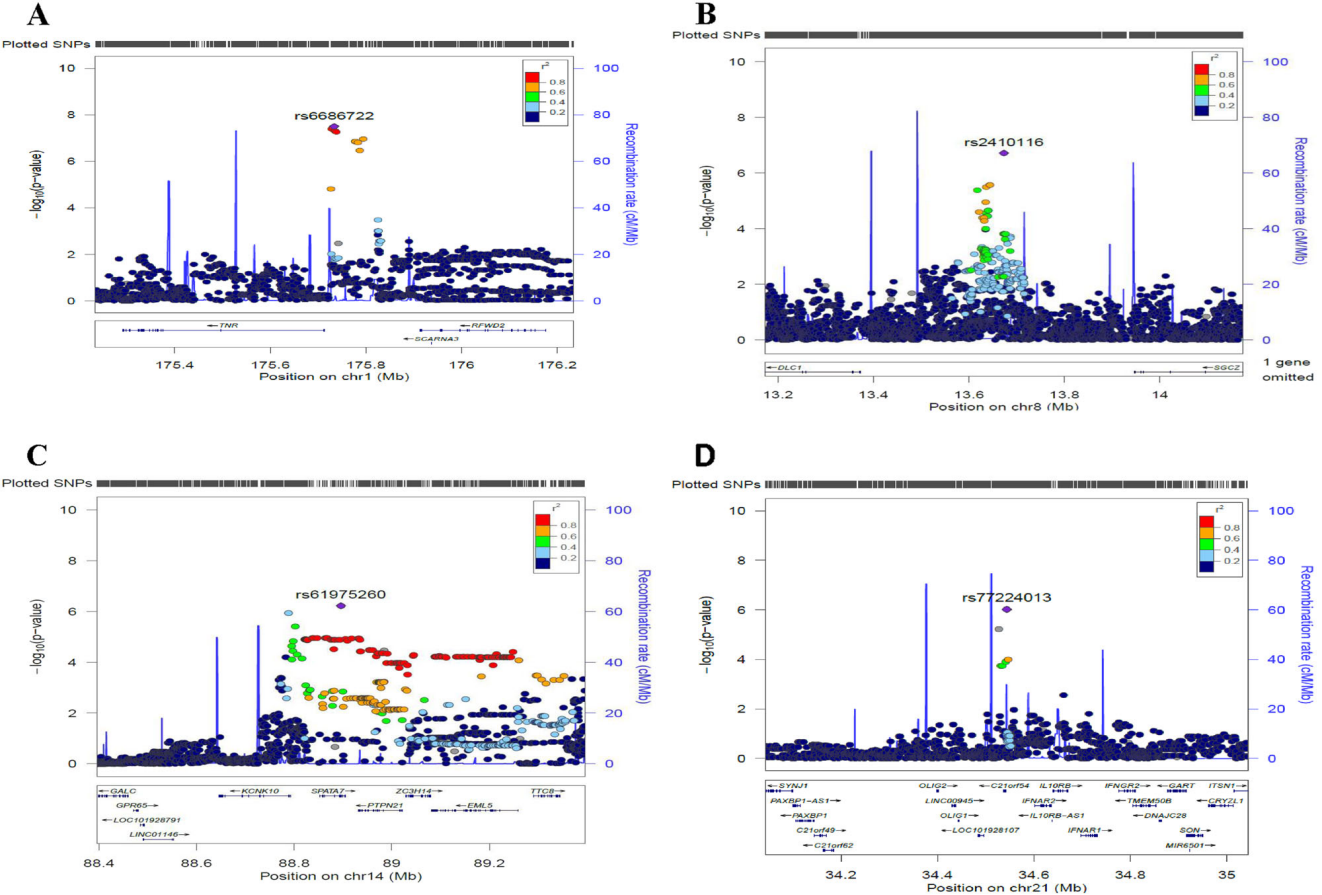
Regional association plots showing the four top GWAS SNPs in ADHD. The most significant SNPs **a** (rs6686722), **b** (rs2410116), **c** (rs61975260) and **d** (rs77224013) are presented as purple diamonds. Genetic recombination rates (cM/Mb) are shown with blue lines (spike)

The Q–Q plot for the association analysis in 480 ADHD cases and 1208 controls showed a slight inflation of *p* values (*λ* = 1.08) relative to expectation under the null distribution (Fig. 2). This lies within acceptable limits (*λ* = 1.01–1.11) and the distribution is skewed at the extreme tail of low *p* values, as expected. As can be seen from the Manhattan plot (Fig. 3) and Table 1, a significant association between ADHD and rs6686722, mapped 22.8 kbp upstream of Tenascin R gene (*TNR*), was observed (*p* = 3.15E−08). The imputation quality of rs6686722 is very high (*r*^2^ = 0.98). Interestingly, and as presented in the regional association plot (Fig. 4), ten SNPs within this region were either significantly associated with ADHD or showed a strong trend towards statistical association, with *p* values ⩽ 3.48E−07.

**Table 1:** Results of the ADHD case–control genome-wide statistical association analysis for the top 20 SNPs

Chr	SNP	BP	A1*	OR	L95	U95	*p* Value	Nearest gene
1	rs6686722	175733963	T	0.4167	0.3056	0.5682	3.15E−08	22.8 kbp upstream of *TNR*
8	rs2410116	13673447	A	0.4991	0.3814	0.6531	4.06E−07	300.2 kbp upstream of *DLC1*
14	rs61975260	88895941	G	0.494	0.3745	0.6516	5.97E−07	*SPATA7*
21	rs77224013	34543845	A	3.869	2.252	6.647	9.64E−07	58.4 kbp upstream of *IFNAR2*
4	rs28612433	25264373	T	0.5385	0.4202	0.6902	1.01E−06	PI4K2B
15	rs4778174	27969566	A	0.5353	0.4164	0.6881	1.07E−06	30.5 kbp downstream of OCA2
19	rs35624673	8134616	T	0.5497	0.4299	0.7029	1.83E−06	*FBN3*
21	rs2015560	26028890	G	0.3596	0.2362	0.5475	1.86E−06	–
11	rs10767556	26623713	G	2.029	1.509	2.728	2.80E−06	ANO3
11	rs28609353	55651658	C	0.4698	0.3411	0.6471	3.74E−06	*TRIM51*
2	rs4673294	205189083	G	0.5462	0.4209	0.7089	5.43E−06	221.5 kbp upstream of *PARD3B*
21	rs112686226	34527379	G	2.903	1.831	4.602	5.78E−06	74.9 kbp upstream of *IFNAR2*
8	rs13439086	8374246	C	2.162	1.549	3.018	5.91E−06	198.9 kbp upstream of *SGK223*
13	rs9545903	82446913	C	1.747	1.37	2.228	6.91E−06	–
1	rs1172198	205662718	A	1.699	1.344	2.149	9.53E−06	*SLC45A3*/*NUCKS1*
9	rs7035982	27417407	A	1.757	1.368	2.257	1.01E−05	*MOB3B*
9	rs35289513	18263813	G	3.038	1.854	4.978	1.03E−05	210.3 kbp upstream of *ADAMTSL1*
6	rs4615440	963496	G	1.781	1.378	2.302	1.05E−05	*LOC285768*
3	rs938524	136521208	G	0.5791	0.4538	0.739	1.13E−05	*SLC35G2*/*STAG1*
18	rs2733140	28363540	T	0.5769	0.4511	0.7378	1.18E−05	250.2 kbp downstream *DSC3*

*Chr* chromosome, *BP* base pair position, * Allele 1, *OR* dds ratio, *L95* lower confidence intervals, *U95* upper confidence intervals, *–* gene desert region,

Three other genomic loci also showed strong trends towards association (*p* values ranging from 9.647E−07 to 4.06E−07; Table 1). The first of these is rs2410116 (*p* = 4.06E−07), which is mapped into a gene desert region (Fig. 4) 300.2 kbp upstream of the gene encoding Rho GTPase Activating Protein (also known as deleted in liver cancer; *DLC1*). The second SNP is rs61975260 (*p* = 5.972E−07), which is mapped to the spermatogenesis associated 7 gene (*SPATA7*). This suggestive association signal is located within a subregion of Chr14 (88788507–89355721) that comprises several genes including *SPATA7, ZC3H14, PTPN21, EML5* and TTC8. Some of these genes, such as *SPATA7* have been implicated in psychiatric conditions, including schizophrenia.

The third of these SNPs is rs77224013 (*p* = 9.647E−07) which is mapped 58.4 kbp upstream of the Interferon Alpha and Beta Receptor Subunit 2 gene (*IFNAR2*). This region of the genome is enriched for genes that function in the immune system (Fig. 4). In addition to the above SNPs, 108 other genomic variants displayed suggestive evidence of association (Table 1 and Supplementary Table 1) with *p* values ⩽ 9.91E−05 to 1.0E−06. We also explored replication of our results within the publically available database of the PGC-iPSYCH meta-analysis^25^. Supplementary Table 1 lists the comparable *p* value within the PGC-iPSYCH meta-analysis against SNPs with *p* values ⩽ 9.91E−05 in the current dataset. Notably, our leading SNP rs6686722 tends towards a nominally significant association in the PGC-iPSCYH meta-analysis (*p* = 0.07). Further, five SNPs from the current study were nominally significant in the PGC-iPSYCH meta-analysis (Supplementary Table 1).

## Discussion

Here, we report the results of a GWAS of 480 probands with childhood ADHD and 1208 controls. PGRS analysis revealed that our sample replicated the published genetic risk profile for ADHD. In fact, PGRS of ADHD, but not other major psychiatric disorders (ASD, SCZ, BP and MDD), explained 3.25% of variance in ADHD case–control status in our cohort (*p* = 7.6E−15). These data demonstrate that the current dataset is enriched for genetic risk for ADHD and thus makes a worthwhile contribution to the international GWAS effort.

Genome-wide association analysis further revealed a significant association with rs6686722 that survived the stringent GWAS correction for multiple comparisons (*p* = 3.15E−08). This SNP is located 22.8 kbp upstream of the Tenascin R (*TNR*) gene. The *TNR* gene is a member of the Tenascin family of the neural extracellular matrix glycoproteins and is highly expressed in the central nervous system^39^. *TNR* is known to function in biological processes such as neural cell adhesion, neurite outgrowth and modulation of sodium channel function^39,40^ that have been implicated in the aetiology of psychiatric disorders. In addition, *TNR* interacts with Fibronectin 1 (*FN1)*, the latter being involved in cell adhesion and migration processes including embryogenesis. This interaction may modulate the adhesive properties of *TNR* during synapse maintenance, a process that is suggested as a risk mechanism for complex psychiatric disorders^41^. Moreover, genetic association studies have implicated the genomic region where *TNR* is located in several brain disorders including schizophrenia, Alzheimer’s disease, narcolepsy and neurological sleep disorder^42^. Further, an additional SNP (rs875326) mapped 2.5 kbp at the 3′untranslated region of the *TNR* was reported to marginally associate with drug response in schizophrenia^43^. Although indirect, our *TNR* ADHD–GWAS finding, combined with findings reported for other psychiatric disorders, provides tentative support for a role of *TNR* in the aetiology of psychiatric conditions.

The second top SNP in our ADHD–GWAS analysis is mapped 300.2 kbp upstream of the Rho GTPase Activating Protein (also known as *DLC1*). Recent studies have revealed common genetic susceptibilities to ADHD and smoking behaviour^44^. Adults with ADHD have higher rates of substance abuse, including higher rates of tobacco smoking^44^. In this context, a recent GWAS analysis of nicotine dependence reported genome-wide significant association with rs289519 (mapped to *DLC1*)^45^. Analysis of rare CNVs across two independent studies of ASD^46,47^, identified rare exonic loss within *DLC1* as risk variants for the disorder. Specifically, Prasad et al.^46^ identified one ASD individual who possessed a rare CNV deletion of 22 kbp across a non-specified exonic region of *DLC1*. Furthermore, Woodbury-Smith et al.^47^ identified a 25 kbp deletion encompassing exon nine on *DLC1* in two unrelated ASD-affected individuals. The above lines of evidence suggest that *DLC1* is a candidate gene worthy of further investigation as a potential susceptibility locus for ADHD and its comorbid disorders (either ASD or substance abuse).

An additional subthreshold association with ADHD was observed for rs61975260 (*p* = 5.972E−07) of the spermatogenesis associated 7 (*SPATA7*) gene which is expressed in the retina and cerebellum. This gene functions in the localisation of retinitis pigmentosa GTPase regulator interacting protein 1 to the photoreceptor connecting cilium (CC), as well as protein trafficking across the CC. Processing speed is a cognitive function that is compromised in psychiatric conditions including ADHD^48,49^. In this context, GWAS analysis^49^ of information processing speed measured using digit symbol, simple reaction time (RT), and 2 and 4-choice RT showed suggestive evidence of association between DNA variation in *SPATA7* and 2-choice RT (*p* = 2.71E−06).

Finally, suggestive evidence of association between ADHD and rs77224013 (9.65E−07) was also observed. Rs77224013 is located 58.4 and 94.8 kbp upstream of the immune cytokine receptors interferon alpha and beta receptor subunit 2 (*IFNAR2*) and interleukin 10 receptor subunit beta (*IL10RB*), respectively. Other immune modulator genes such as *IL10RB*-*As1* and *IFNAR1* also map to this region. Immune imbalance has been suggested as a predisposing factor for ADHD in genetically susceptible individuals^50^. Significantly increased transmission of IL-*1Ra 4*-repeat allele and decreased transmission of 2-repeat allele of a variable number tandem repeat polymorphism to ADHD-affected children was reported by Segman et al.^51^ Further, immune dysregulation is supported by the finding that ADHD individuals have four times higher concentrations of Interleukins (*IL*-*1* and *IL*-*6*) than typically developing children^52^. This led Verlaet et al.^50^ to hypothesise that “overproduction of these cytokines could lead to chronic inflammation in brain tissues”. This is consistent with brain anomalies in children with ADHD. For example, Nopoulous et al.^53^ reported increased frequency of gray-matter heterotropia (ectopic nodules of neurons) and posterior fossa abnormalities in ADHD patients compared to controls. Further, reduced cortical volume associated with reduced surface area and gyrification were also reported in ADHD compared to controls^54^. However, the correlation between overproduction of cytokines and brain anomalies in ADHD requires further evidence to establish a causal link.

Our sample has provided replication evidence for some of the results arising from the recent and largest ADHD meta-analysis by the PGC-IPSYCH consortia^25^. For example, we report a nominal association with rs281324 (*p* = 0.045), mapped to intron 3 of semaphorin 6D (SEMA6D) gene which sits within 70 kbp genomic region of significant LD. Members of the semaphorin gene family have been implicated as inhibitors or chemo-repellents in axon pathfinding and fasciculation and branching. More recently, Klein et al.^55^, examined if the genetic risk markers (reported by the PGC meta-analysis) mediate alteration in brain structure. They observed that rs281323 (in perfect LD with rs281324) is significantly associated with increased risk for ADHD and putamen volume. Further, the SEMA6D rs281323 is strongly associated with the expression level of SEMA6D^55^. Together, these finding clearly implicate SEMA6D as a susceptibility locus for ADHD.

Finally, it is important to emphasise that our study has a number of major limitations. First, the discovery sample is small and has limited statistical power to detect a reliable genome-wide association signal. Further, our leading SNP association for *TNR* (rs6686722) is not significant within the much larger PGC-IPSYCH meta-analysis of ADHD (*p* = 0.07). Notwithstanding these limitations, our ADHD cohort is clearly enriched for genetic risk for ADHD, as evinced by our strong PGRS results for ADHD (but not the other major psychiatric disorders). As such, our study therefore makes an important contribution to the international genetics effort for ADHD.

## Supplementary information

Supplimentry Table 1
