# Supplementary material for: A case–control genome-wide association study of ADHD discovers a novel association with the tenascin R (TNR) gene
Source: Transl Psychiatry. 2018 Dec 18;8:284. doi: 10.1038/s41398-018-0329-x (PMC6298965; doi:10.1038/s41398-018-0329-x)
Supplement: Supplementary file 1 — Supplimentry Table 1 [file 41398_2018_329_MOESM1_ESM.docx]

**Supplementary Table 1**: ADHD-GWAS analysis showing significant association with rs6686722 and suggestive evidence of SNPs associations

(p=⩽9.99 E−05). P-value for these SNPs reported by the PGC-ADHD meta-analysis is also presented.

| Chr | SNP | BP | A1 | OR | SE | L95 | U95 | STAT | P-value  (our data) | P-value (PGC-iPSYCH GWAS meta-analysis) |
| --- | --- | --- | --- | --- | --- | --- | --- | --- | --- | --- |
| 1 | rs6686722 | 175733963 | T | 0.4167 | 0.1582 | 0.3056 | 0.5682 | −5.533 | 3.15E−08 | 0.07018 |
| 8 | rs2410116 | 13673447 | A | 0.4991 | 0.1372 | 0.3814 | 0.6531 | −5.066 | 4.06E−07 | 0.8657 |
| 14 | rs61975260 | 88895941 | G | 0.494 | 0.1413 | 0.3745 | 0.6516 | −4.992 | 5.97E−07 | 0.186 |
| 21 | rs77224013 | 34543845 | A | 3.869 | 0.2762 | 2.252 | 6.647 | 4.899 | 9.65E−07 | 0.1968 |
| 4 | rs28612433 | 25264373 | T | 0.5385 | 0.1266 | 0.4202 | 0.6902 | −4.89 | 1.01E−06 | 0.7564 |
| 15 | rs4778174 | 27969566 | A | 0.5353 | 0.1281 | 0.4164 | 0.6881 | −4.878 | 1.07E−06 | 0.3883 |
| 19 | rs35624673 | 8134616 | T | 0.5497 | 0.1254 | 0.4299 | 0.7029 | −4.771 | 1.84E−06 | 0.7058 |
| 21 | rs2015560 | 26028890 | G | 0.3596 | 0.2145 | 0.2362 | 0.5475 | −4.768 | 1.86E−06 | 0.1995 |
| 11 | rs10767556 | 26623713 | G | 2.029 | 0.151 | 1.509 | 2.728 | 4.685 | 2.8E−06 | 0.09156 |
| 11 | rs28609353 | 55651658 | C | 0.4698 | 0.1633 | 0.3411 | 0.6471 | −4.625 | 3.74E−06 | NG |
| 2 | rs4673294 | 205189083 | G | 0.5462 | 0.133 | 0.4209 | 0.7089 | −4.547 | 5.43E−06 | 0.1144 |
| 21 | rs112686226 | 34527379 | G | 2.903 | 0.235 | 1.831 | 4.602 | 4.534 | 5.78E−06 | NG |
| 8 | rs13439086 | 8374246 | C | 2.162 | 0.1702 | 1.549 | 3.018 | 4.529 | 5.91E−06 | 0.6952 |
| 13 | rs9545903 | 82446913 | C | 1.747 | 0.1241 | 1.37 | 2.228 | 4.497 | 6.91E−06 | 0.7975 |
| 1 | rs1172198 | 205662718 | A | 1.699 | 0.1197 | 1.344 | 2.149 | 4.428 | 9.53E−06 | 0.01342 |
| 9 | rs7035982 | 27417407 | A | 1.757 | 0.1277 | 1.368 | 2.257 | 4.414 | 1.01E−05 | 0.08063 |
| 9 | rs35289513 | 18263813 | G | 3.038 | 0.2519 | 1.854 | 4.978 | 4.411 | 1.03E−05 | 0.04082 |
| 6 | rs4615440 | 963496 | G | 1.781 | 0.1309 | 1.378 | 2.302 | 4.407 | 1.05E−05 | 0.323 |
| 3 | rs938524 | 136521208 | G | 0.5791 | 0.1244 | 0.4538 | 0.739 | −4.391 | 1.13E−05 | 0.1163 |
| 18 | rs2733140 | 28363540 | T | 0.5769 | 0.1255 | 0.4511 | 0.7378 | −4.382 | 1.18E−05 | 0.6157 |
| 2 | rs11682209 | 156801077 | T | 0.5087 | 0.155 | 0.3754 | 0.6892 | −4.361 | 1.29E−05 | 0.909 |
| 7 | rs181385572 | 46991385 | A | 2.915 | 0.248 | 1.793 | 4.74 | 4.315 | 1.6E−05 | NG |
| 9 | rs57380099 | 10169291 | G | 1.88 | 0.1465 | 1.411 | 2.506 | 4.312 | 1.62E−05 | 0.9868 |
| 5 | rs72805238 | 112539604 | A | 2.104 | 0.1729 | 1.499 | 2.952 | 4.303 | 1.69E−05 | 0.9279 |
| 1 | rs55649783 | 39109615 | T | 0.5191 | 0.1525 | 0.385 | 0.6999 | −4.3 | 1.71E−05 | 0.4805 |
| 13 | rs985288 | 22787338 | G | 1.773 | 0.1333 | 1.365 | 2.302 | 4.296 | 1.74E−05 | 0.5726 |
| 15 | rs76805726 | 82486236 | T | 0.3672 | 0.2352 | 0.2316 | 0.5822 | −4.26 | 2.05E−05 | 0.991 |
| 13 | rs61945322 | 22786670 | G | 1.835 | 0.1433 | 1.386 | 2.43 | 4.236 | 2.28E−05 | 0.5898 |
| 10 | rs185060603 | 105258139 | A | 3.326 | 0.2852 | 1.902 | 5.818 | 4.214 | 2.51E−05 | 0.4402 |
| 11 | rs6588954 | 107115380 | C | 0.5966 | 0.1226 | 0.4691 | 0.7586 | −4.213 | 2.52E−05 | 0.1733 |
| 3 | rs13061236 | 62719783 | G | 1.89 | 0.1511 | 1.405 | 2.541 | 4.212 | 2.53E−05 | 0.4395 |
| 12 | rs34195345 | 3245990 | T | 0.4948 | 0.1675 | 0.3563 | 0.6871 | −4.2 | 2.67E−05 | 0.5409 |
| 14 | rs77188309 | 96219460 | A | 0.4127 | 0.2107 | 0.273 | 0.6237 | −4.2 | 2.67E−05 | 0.2316 |
| 12 | rs80268361 | 26266708 | C | 3.225 | 0.2794 | 1.865 | 5.578 | 4.191 | 2.78E−05 | 0.3608 |
| 3 | rs34937600 | 9328349 | G | 2.342 | 0.2033 | 1.572 | 3.488 | 4.186 | 2.83E−05 | 0.09545 |
| 1 | rs670074 | 96056162 | T | 1.756 | 0.1346 | 1.349 | 2.286 | 4.185 | 2.85E−05 | 0.05171 |
| 12 | rs6581726 | 67362499 | T | 0.5859 | 0.1278 | 0.4561 | 0.7526 | −4.185 | 2.85E−05 | 0.2836 |
| 15 | rs7167686 | 70618956 | A | 1.683 | 0.1244 | 1.319 | 2.148 | 4.183 | 2.88E−05 | 0.3408 |
| 1 | rs1539129 | 3484237 | T | 0.5393 | 0.1486 | 0.4031 | 0.7216 | −4.156 | 3.25E−05 | NG |
| 6 | rs9388250 | 123816195 | A | 0.5414 | 0.1478 | 0.4053 | 0.7233 | −4.153 | 3.29E−05 | 0.5558 |
| 16 | rs2654454 | 80352195 | C | 1.957 | 0.162 | 1.425 | 2.689 | 4.145 | 3.39E−05 | 0.4255 |
| 7 | rs73692920 | 49740344 | A | 0.3649 | 0.2439 | 0.2262 | 0.5886 | −4.133 | 3.59E−05 | 0.2383 |
| 15 | rs79069161 | 27734093 | T | 0.4693 | 0.184 | 0.3272 | 0.6732 | −4.11 | 3.96E−05 | NG |
| 6 | rs1871859 | 151898506 | T | 2.215 | 0.1936 | 1.516 | 3.237 | 4.108 | 3.99E−05 | 0.6244 |
| 7 | rs13228276 | 48140799 | G | 0.5981 | 0.1252 | 0.468 | 0.7644 | −4.107 | 4.01E−05 | 0.4656 |
| 16 | rs17269910 | 52268819 | G | 2.063 | 0.1764 | 1.46 | 2.915 | 4.106 | 4.03E−05 | 0.4596 |
| 11 | rs7106053 | 17458730 | G | 0.5838 | 0.1313 | 0.4514 | 0.7552 | −4.098 | 4.16E−05 | 0.5369 |
| 4 | rs10049978 | 25523522 | C | 0.4884 | 0.1749 | 0.3466 | 0.688 | −4.098 | 4.17E−05 | 0.8092 |
| 13 | rs9510037 | 22778228 | G | 1.741 | 0.1355 | 1.335 | 2.27 | 4.09 | 4.31E−05 | 0.8069 |
| 14 | rs11846171 | 101624406 | A | 1.619 | 0.1178 | 1.285 | 2.039 | 4.09 | 4.31E−05 | 0.8329 |
| 15 | rs4145152 | 54255505 | C | 1.681 | 0.1273 | 1.31 | 2.158 | 4.082 | 4.47E−05 | 0.02564 |
| 10 | rs7092999 | 102375258 | A | 0.6013 | 0.1248 | 0.4708 | 0.7678 | −4.078 | 4.55E−05 | 0.3402 |
| 2 | rs10931434 | 153881453 | G | 2.084 | 0.1802 | 1.464 | 2.967 | 4.075 | 4.6E−05 | 0.8985 |
| 10 | rs56024660 | 127845104 | A | 0.4633 | 0.1889 | 0.32 | 0.6709 | −4.073 | 4.64E−05 | 0.2834 |
| 1 | rs72773758 | 247660504 | G | 1.88 | 0.1552 | 1.387 | 2.549 | 4.067 | 4.77E−05 | 0.6282 |
| 6 | rs9375001 | 121416715 | C | 1.839 | 0.1499 | 1.371 | 2.467 | 4.063 | 4.84E−05 | 0.7815 |
| 13 | rs9541989 | 70181549 | G | 0.5126 | 0.1648 | 0.3711 | 0.7081 | −4.054 | 5.04E−05 | 0.2539 |
| 6 | rs9369238 | 40772971 | T | 0.5513 | 0.147 | 0.4133 | 0.7354 | −4.05 | 5.12E−05 | 0.6653 |
| 10 | rs7098019 | 16683092 | T | 0.5947 | 0.1286 | 0.4622 | 0.7651 | −4.043 | 5.29E−05 | NG |
| 2 | rs112916620 | 26170228 | T | 2.237 | 0.1992 | 1.514 | 3.305 | 4.042 | 5.31E−05 | NG |
| 1 | rs10737300 | 173385186 | T | 0.5396 | 0.1528 | 0.3999 | 0.728 | −4.037 | 5.41E−05 | 0.5098 |
| 14 | rs7141004 | 57038353 | C | 1.654 | 0.1247 | 1.295 | 2.111 | 4.035 | 5.47E−05 | 0.4983 |
| 19 | rs62122033 | 35214675 | C | 0.5837 | 0.1335 | 0.4493 | 0.7582 | −4.034 | 5.49E−05 | 0.9975 |
| 1 | rs61103655 | 154630066 | T | 1.764 | 0.1409 | 1.339 | 2.326 | 4.031 | 5.56E−05 | 0.06218 |
| 20 | rs6013564 | 51535828 | A | 0.5916 | 0.1304 | 0.4582 | 0.7638 | −4.027 | 5.64E−05 | 0.1622 |
| 20 | rs1856511 | 15962055 | T | 0.6139 | 0.1212 | 0.4841 | 0.7785 | −4.026 | 5.68E−05 | 0.6083 |
| 1 | rs6678710 | 177317366 | T | 1.682 | 0.1293 | 1.306 | 2.167 | 4.023 | 5.74E−05 | 0.1404 |
| 9 | rs28668478 | 135064450 | G | 0.6106 | 0.1228 | 0.48 | 0.7767 | −4.018 | 5.86E−05 | 0.01884 |
| 11 | rs10894594 | 132501022 | C | 1.8 | 0.1463 | 1.351 | 2.398 | 4.017 | 5.89E−05 | 0.3367 |
| 7 | rs36008132 | 42148530 | G | 0.573 | 0.139 | 0.4364 | 0.7525 | −4.005 | 6.21E−05 | 0.2163 |
| 14 | rs734584 | 40674440 | G | 2.422 | 0.2212 | 1.57 | 3.737 | 3.999 | 6.35E−05 | 0.105 |
| 7 | rs35791057 | 55060497 | A | 2.41 | 0.2199 | 1.566 | 3.708 | 3.999 | 6.35E−05 | 0.2875 |
| 18 | rs2644254 | 34206440 | A | 0.5497 | 0.1499 | 0.4097 | 0.7374 | −3.992 | 6.55E−05 | 0.8159 |
| 1 | rs1417759 | 58053427 | G | 1.753 | 0.1407 | 1.331 | 2.31 | 3.99 | 6.61E−05 | 0.6333 |
| 8 | rs4740005 | 80738751 | T | 0.6141 | 0.1223 | 0.4832 | 0.7805 | −3.986 | 6.71E−05 | 0.806 |
| 7 | rs844743 | 71543905 | T | 0.6164 | 0.1214 | 0.4859 | 0.782 | −3.986 | 6.73E−05 | 0.1554 |
| 19 | rs7246572 | 54186902 | A | 0.5131 | 0.1675 | 0.3695 | 0.7124 | −3.984 | 6.77E−05 | 0.7454 |
| 10 | rs11202685 | 89986002 | A | 2.768 | 0.2557 | 1.677 | 4.569 | 3.982 | 6.85E−05 | NG |
| 19 | rs7255545 | 4724734 | A | 1.668 | 0.1285 | 1.296 | 2.145 | 3.981 | 6.86E−05 | NG |
| 14 | rs2626446 | 42902703 | G | 0.5878 | 0.1337 | 0.4523 | 0.7638 | −3.976 | 7.02E−05 | 0.9548 |
| 14 | rs4379974 | 34684484 | G | 0.5516 | 0.1498 | 0.4113 | 0.7398 | −3.972 | 7.11E−05 | 0.1341 |
| 3 | rs952389 | 138924550 | G | 0.5841 | 0.1355 | 0.4479 | 0.7617 | −3.97 | 7.2E−05 | 0.3557 |
| 4 | rs13118422 | 177384510 | C | 0.5946 | 0.1311 | 0.4598 | 0.7688 | −3.964 | 7.36E−05 | 0.05408 |
| 15 | rs141976159 | 83376056 | T | 0.3996 | 0.2314 | 0.2539 | 0.6289 | −3.964 | 7.37E−05 | 0.9124 |
| 4 | rs7692570 | 83209493 | T | 1.619 | 0.1216 | 1.275 | 2.055 | 3.96 | 7.49E−05 | 0.8868 |
| 19 | rs10164318 | 33459298 | T | 1.672 | 0.1299 | 1.296 | 2.157 | 3.959 | 7.53E−05 | 0.3033 |
| 13 | rs12874870 | 82529830 | C | 1.968 | 0.1711 | 1.408 | 2.753 | 3.958 | 7.56E−05 | 0.1384 |
| 2 | rs35618657 | 124480145 | C | 1.721 | 0.1372 | 1.315 | 2.252 | 3.958 | 7.56E−05 | 0.8612 |
| 5 | rs60714766 | 156602589 | T | 1.736 | 0.1394 | 1.321 | 2.282 | 3.957 | 7.6E−05 | 0.278 |
| 3 | rs12497578 | 152556473 | G | 0.4652 | 0.1935 | 0.3183 | 0.6797 | −3.955 | 7.66E−05 | 0.04112 |
| 13 | rs9532007 | 37732909 | A | 0.5418 | 0.1551 | 0.3997 | 0.7343 | −3.951 | 7.79E−05 | 0.07581 |
| 4 | rs11099615 | 84607459 | G | 0.5422 | 0.155 | 0.4002 | 0.7347 | −3.949 | 7.83E−05 | 0.4017 |
| 3 | rs923578 | 72620595 | G | 0.3438 | 0.2704 | 0.2024 | 0.5842 | −3.948 | 7.88E−05 | 0.4549 |
| 1 | rs12093301 | 177188011 | G | 1.772 | 0.1451 | 1.334 | 2.356 | 3.944 | 8.01E−05 | 0.251 |
| 15 | rs731597 | 87271959 | T | 1.675 | 0.131 | 1.296 | 2.166 | 3.939 | 8.19E−05 | 0.2913 |
| 10 | rs7075459 | 63908042 | C | 2.043 | 0.1814 | 1.432 | 2.916 | 3.938 | 8.23E−05 | 0.8515 |
| 11 | rs2514268 | 91318007 | A | 0.6135 | 0.1241 | 0.481 | 0.7825 | −3.935 | 8.3E−05 | 0.1229 |
| 12 | rs11613377 | 89383659 | A | 0.3508 | 0.2664 | 0.2081 | 0.5912 | −3.933 | 8.39E−05 | 0.4093 |
| 10 | rs112237588 | 32508615 | A | 0.4899 | 0.1815 | 0.3432 | 0.6992 | −3.931 | 8.44E−05 | 0.3206 |
| 15 | rs11071435 | 59671341 | A | 0.6112 | 0.1254 | 0.478 | 0.7815 | −3.927 | 8.61E−05 | 0.3206 |
| 8 | rs2139583 | 61090507 | A | 0.6015 | 0.1295 | 0.4667 | 0.7753 | −3.925 | 8.67E−05 | 0.1973 |
| 5 | rs35230338 | 152033773 | C | 1.701 | 0.1355 | 1.304 | 2.219 | 3.92 | 8.84E−05 | 0.05535 |
| 20 | rs74171674 | 18209177 | C | 2.156 | 0.1961 | 1.468 | 3.167 | 3.918 | 8.92E−05 | 0.923 |
| 8 | rs139478003 | 19337302 | A | 2.916 | 0.2735 | 1.706 | 4.985 | 3.913 | 9.11E−05 | NG |
| 13 | rs9590116 | 95564854 | G | 1.688 | 0.1338 | 1.298 | 2.193 | 3.912 | 9.14E−05 | 0.445 |
| 13 | rs9574993 | 82485367 | T | 0.6103 | 0.1263 | 0.4765 | 0.7818 | −3.909 | 9.27E−05 | 0.1406 |
| 11 | rs117598608 | 33831646 | T | 0.4371 | 0.2118 | 0.2887 | 0.662 | −3.908 | 9.32E−05 | 0.2094 |
| 3 | rs12634712 | 45252125 | A | 0.6084 | 0.1274 | 0.474 | 0.7809 | −3.901 | 9.58E−05 | 0.1196 |
| 13 | rs927550 | 22786891 | C | 1.691 | 0.1346 | 1.299 | 2.201 | 3.901 | 9.58E−05 | 0.487 |
| 3 | rs2574728 | 11611734 | C | 0.6092 | 0.1272 | 0.4748 | 0.7818 | −3.895 | 9.83E−05 | 0.6003 |
| 9 | rs1231375 | 25755952 | T | 0.5263 | 0.1649 | 0.381 | 0.7271 | −3.893 | 9.9E−05 | 0.8952 |
| 1 | rs7544386 | 83485083 | T | 1.749 | 0.1436 | 1.32 | 2.317 | 3.893 | 9.91E−05 | 0.6974 |

NG: Not Genotyped
